# Male fertility potential alteration in rheumatic diseases: a systematic review

**DOI:** 10.1590/S1677-5538.IBJU.2014.0595

**Published:** 2016

**Authors:** Bruno Camargo Tiseo, Marcello Cocuzza, Eloisa Bonfá, Miguel Srougi, A Clovis

**Affiliations:** 1 Departamento de Urologia da Faculdade de Medicina da Universidade de São Paulo, Brasil;; 2 Divisão de Reumatologia da Faculdade de Medicina da Universidade de São Paulo, Brasil;; 3 Unidade de Reumatologia Pediátrica do Departamento de Pediatria da Faculdade de Medicina da Universidade de São Paulo, Brasil

**Keywords:** Rheumatic Diseases, Fertility, Infertility, Male

## Abstract

**Background:**

Improved targeted therapies for rheumatic diseases were developed recently resulting in a better prognosis for affected patients. Nowadays, patients are living longer and with improved quality of life, including fertility potential. These patients are affected by impaired reproductive function and the causes are often multifactorial related to particularities of each disease. This review highlights how rheumatic diseases and their management affect testicular function and male fertility.

**Materials and Methods:**

A systematic review of literature of all published data after 1970 was conducted. Data was collected about fertility abnormalities in male patients with systemic lupus erythematosus, rheumatoid arthritis, dermatomyositis, ankylosing spondylitis, Behçet disease and gout. Two independent researchers carried out the search in online databases.

**Results:**

A total of 19 articles were included addressing the following diseases: 7 systemic lupus erythematosus, 6 Behçet disease, 4 ankylosing spondylitis, 2 rheumatoid arthritis, 2 dermatomyositis and one gout. Systemic lupus erythematosus clearly affects gonadal function impairing spermatogenesis mainly due to antisperm antibodies and cyclophosphamide therapy. Behçet disease, gout and ankylosing spondylitis patients, including those under anti-TNF therapy in the latter disease, do not seem to have reduced fertility whereas in dermatomyositis, the fertility potential is hampered by disease activity and by alkylating agents. Data regarding rheumatoid arthritis is scarce, gonadal dysfunction observed as consequence of disease activity and antisperm antibodies.

**Conclusions:**

Reduced fertility potential is not uncommon. Its frequency and severity vary among the different rheumatic diseases. Permanent infertility is rare and often associated with alkylating agent therapy.

## INTRODUCTION

There are 1.3 million adults affected by rheumatoid arthritis (RA) and up to 322.000 by systemic lupus erythematosus (SLE) in United States (1). Improved targeted therapies for rheumatic diseases have been developed recently resulting in better prognosis. In this context health-related quality of life became a major concern, including reproductive issues (2).

Decreased fertility potential is not unusual among patients of both genders with rheumatic diseases, particularly in women (3, 4) with many articles addressing in RA, SLE, ankylosing spondylitis (AS), dermatomyositis (DM), Behçet disease (BD) and gout (5-8). Drug treatment is probably the main factor for gonadal dysfunction (9). Some drugs can cause reversible infertility, such as nonsteroidal antiinflammatory drugs in women and sulfasalazine/methotrexate in men whereas irreversible infertility is occasionally observed after treatment with alkylating agents (cyclophosphamide-CYC and chlorambucil) in both genders (10). When fertility is an issue, alkylating agents should be used at lowest possible dose and alternative therapies (such as azathioprine or mycophenolate mofetil) must be considered.

The reproduction potential of these male patients is impaired by the disease directly in the testicular tissue or by immunosuppressive therapy (11). The evaluation of male subjects should rely on careful medical history, complete physical examination, semen analysis and sexual hormone profile.

The objective of this systematic review of the literature on rheumatic disease male fertility potential is to provide a better understanding to urologists, andrologists, infertility specialists and rheumatologists of the underlying contributing factors involved, as well as discuss how fertility potential is endangered by diseases management.

## SEARCH STRATEGY AND SELECTION CRITERIA

It was conducted a computerized search of English and non-English language articles published after 1970 listed in the electronic databases of SCOPUS, PUBMED/MEDLINE and Cochrane Library. Two independent researchers (MC, BT) conducted the search during May-July 2014. The following terms were used: ‘systemic lupus erythematosus’, ‘ankylosing spondylitis’, ‘dermatomyositis’, ‘rheumatoid arthritis’, ‘Behçet disease’, ‘gout’, ‘male infertility’, ‘pregnancy rate’, ‘sperm’, ‘semen’, ‘spermatozoa’, ‘sperm quality’ and ‘rheumatic disease’. The search was performed in English language but articles yielded in other languages were not excluded. The authors graded the abstract of each study identified by the search to determine eligibility. If these criteria remained unclear from the abstract, the full article was retrieved for clarification.

Data extraction was carried out by the investigators using a standardized data collection form with subsequent discussion with all authors. Peer-reviewed observational controlled and non-controlled studies (case–control and cohort studies) were selected. All studies were referral centre-, hospital-or population-based studies. The data collected in the selected articles were all related to fertility abnormalities in male patients with SLE, RA, DM, AS, BD and gout. We excluded articles that were case reports and those that did not evaluated male patients.

## RESULTS

**Figure 1:**
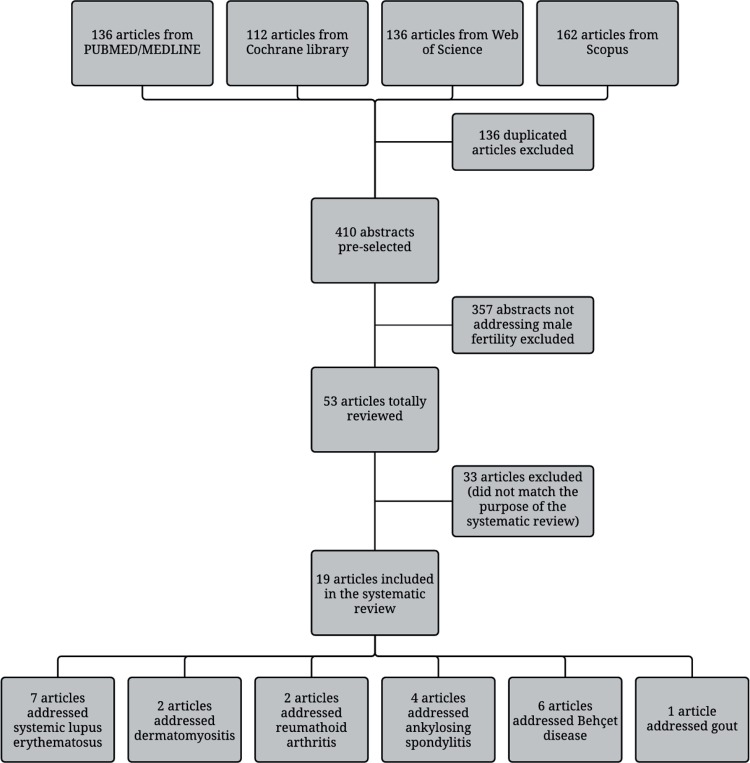
Flow of articles through different phases of the systematic review.

The article flow through the selection phase is summarized in Figure-1. An initial search of online databases yielded 136 publications from PUBMED/MEDLINE, 112 reviews from Cochrane Library, 136 from Web of Science, and 162 from Scopus. After excluding duplicated publications and applying exclusion criteria, 19 relevant articles were included with the following diseases: 7 SLE, 2 DM, 2 RA, 4 AS, 6 BD and one with gout. There was one article evaluating simultaneously two diseases and another addressing three (Figure-1).

### Systemic lupus erythematosus

Publications selected focused on four aspects of male fertility in SLE: gonadal dysfunction, testicular alterations induced by immunosuppressive treatment, presence of anti-sperm antibody and genetic abnormalities (Table-1).

**Table 1:** Systematic lupus erythematosus and male fertility according to gonadal dysfunction, immunosuppressive agents, anti-sperm antibody and sex chromosomes aneuploid.

Author	Year	Study Population	Results	Conclusion
Gonadal dysfunction				

Soares et al. (12)	2007	35 patients with SLE and 35 normal controls	SLE patients had low sperm count, low motile sperm and low normal sperm forms / Sperm abnormalities related to elevated FSH levels	Gonad function is severely affected in male SLE patients due to testicular damage
Suehiro et al. (13)	2008	34 patients with SLE	23% had decreased inhibin B and elevated FSH, 15% had decreased testosterone, 70% sperm analysis alterations, 20% reduced testicular volume	Sertoli cell dysfunction in male SLE affecting inhibin B secretion. It was related to impaired sperm production
Silva et al. (14)	2009	25 patients with SLE and 25 normal controls	20% SLE patients had erectile dysfunction, 36% had elevated FSH and 48% had sperm abnormalities	SLE affects whole male reproductive health, particularly under CYC / No influence of ASA

Testicular damage immunosuppressive agents				

Silva et al. (15)	2002	4 patients with juvenile SLE	1 patient with azoospermia and elevated FSH / 1 patient with severe oligospermia. Both treated with CYC	Alkylating agents may induce testicular damage

Presence of anti-sperm antibody				

D'Cruz et al. (16)	1994	33 patients with SLE, 33 patients with ASA and 20 normal controls	42% patients with SLE had ASA	ASA has high frequency in SLE patients
Shiraishi et al. (17)	2008	32 patients with RA, 14 with BD, 8 with SLE and 80 healthy controls	13% SLE patients had ASA	SLE may induce ASA in patients and affect fertility

Presence of sex chromosomes aneuploid				

Dillon et al. (18)	2012	316 patients with SLE	8 patients with sex chromosomes alterations	Sex chromosomes aneuploid is more common in SLE men and may impact their fertility

**Note: SLE =** systemic lupus erythematosus; **LH =** luteinizing hormone; **FSH =** follicle-stimulating hormone; **CYC =** cyclophosphamide; **RA =** rheumathoid arthritis; **ASA =** antisperm antibody, **BD =** Behçet disease.

A global gonadal function evaluation was performed by our Group (12) assessing sex hormone profile, semen analysis and antisperm analysis (ASA). Thirty-five patients compared to paired controls had lower testicular volumes, total sperm count and total motile sperm count associated with CYC use.

We investigated Sertoli cell function analyzing inhibin B levels and semen abnormalities in SLE patients. Lower inhibin B level was correlated with diminished sperm count, concentration and total motility count and with elevated FSH and LH levels (13). In addition, it was observed that 20% of SLE patients had erectile dysfunction, 36% of testicles were below the normal volume range and 48% had semen analysis abnormalities associated with CYC therapy (14). The same gonadotoxic effect of CYC was also reported in four patients with juvenile SLE (15).

Serum IgG ASA targeting the sperm head and/or midpiece was reported in 15% and antisperm deoxyribonucleic acid antibodies were found in 42% of SLE patients, indicating that autoimmunity is another contributing factor in these patient’s (16). This finding was confirmed in 8 patients evaluated by Shiraishi et al. (17).

Recently, Dillon et al. (18) evaluated the karyotype of 316 men with SLE and 1201 healthy controls. Aneuploidies were evidenced in 2.5% male SLE patients and none in controls. There was three 47, XXY, three patients with mosaic 46, XY/47, XXY, one had 46, XX/47, XXY mosaicism and another one had 46, XX karyotype.

### Dermatomyositis

The two publications addressing DM patient’s fertility are illustrated in Table-2. Moraes et al. (19) evaluated five patients with juvenile DM and compared with 10 age-matched healthy controls regarding testicular volume, sperm analysis, ASA and sex hormone profile. One patient had used CYC with a cumulative dose of 6.6g and experienced transient azoospermia with normalization after 5 years of medication withdrawal. All DM patients had teratospermia, one had ASA and none had abnormal hormone profile.

**Table 2:** – Dermatomyositis and male fertility.

Author	Year	Study Population	Results	Conclusion
Moraes et al. (19)	2008	5 post-pubertal males with DM and 5 healthy controls	100% teratozoospermia / Azoospermia during CYC treatment	No significant difference between patients and normal controls regarding hormonal levels or sperm analysis
Moraes et al. (20)	2010	10 post-pubertal males with DM and 10 healthy controls	Low concentration, low sperm abnormalities, low motile count, reduced testicular volumes in DM patients	DM may affect testicular function and sex hormones levels / Disease activity and CYC may induce gonadal dysfunction

**Note: DM =** dermatomyositis; **CYC =** cyclophosphamide.

A later study investigated 10 adult patients and 10 age-matched healthy controls. DM subjects had lower sperm concentration, lower total motile sperm count and lower normal sperm morphology percentage. Disease activity seemed to be a relevant factor in four patients and CYC in one of them (20).

### Rheumatoid arthritis

Two publications assessed fertility on RA patients (Table-3). Gordon et al. (21) evaluated 31 patients with RA, 33 with AS and 95 healthy controls. Patients with RA had lower serum testosterone levels and higher FSH and LH levels than controls. Ten patients (33%) admitted periods of erectile dysfunction while 15 (50%) also referred decreased libido when suffering from arthritis. Four patients referred difficulty to conceive, among them, two did not seek medical assistance for infertility. Nineteen males had successfully fathered children and the others were still singles.

**Table 3:** Rheumatoid arthritis and male fertility.

Author	Year	Study Population	Results	Conclusion
Gordon et al. (21)	1986	31 patients with RA, 33 with AS patients and 95 normal controls	Low testosterone / Elevated FSH and LH level in patients with RA	Normal pituitary-gonad axis function / Testicular damage by disease activity
Shiraishi et al. (17)	2008	32 men with RA, 14 with BD, 8 with SLE and 80 healthy controls	3% RA patients had ASA	RA may induce ASA in patients and may affect fertility

**Note: RA** = rheumathoid arthritis; **AS =** ankylosing spondylitis; **BD =** Behçet disease; **FSH =** follicle-stimulating hormone; **LH =** luteinizing hormone; **SLE =** systemic lupus erythematosus; **ASA =** antisperm antibody.

In 2008, Shiraishi et al. (17) evaluated 32 RA patients and found one with serum ASA. The patient was 74 years old and the disease onset was at the age of 60 years. He had already 2 children before being diagnosed with the disease so the relation between fertility status and the presence of the antibody could not be addressed nor its relation with the disease.

### Ankylosing spondylitis

Four publications were selected regarding the AS association with male fertility. The major aspects of each paper are summarized in Table-4. A total of 33 AS patients were evaluated in 1986, reporting four patients with erectile dysfunction and 11 with decreased libido. Only one male with AS had an infertile marriage and did not seek for medical assistance. Thirteen patients were singles and all other had constituted their families without problems (21). Varicocele was an additional and frequent finding in AS males (40%), of another cohort, and its impact in male fertility remains to be determined, since only mild sperm abnormalities was observed in these patients (22).

**Table 4:** – Ankylosing spondylitis and male fertility.

Author	Year	Study Population	Results	Conclusion
Gordon et al. (21)	1986	31 patients with RA, 33 with AS patients and 95 normal controls	Normal testosterone / Normal FSH and LH levels / 13% impotence / 39% decreased libido	AS may affect libido and erectile function / No impact in testicular function
Paschou et al. (7)	2009	4 patients treated with infliximab	All patients had fathered at least one child	AS patients treated with anti-TNF seem not to suffer infertility issues
Nukumizu et al. (23)	2012	20 patients with AS and 24 healthy controls	40% of AS patients had varicocele and was associated to teratospermia	Varicocele is frequent in AS and may affect sperm morphology impairing fertility
Almeida et al. (22)	2013	20 patients with AS and 24 healthy controls	Normal sex hormones levels, including inhibin B / Normal seminal parameters	Sertoli cell function was not affected by AS or by anti-TNF therapy

**Note: RA =** rheumathoid arthritis; **AS = a**nkylosing spondylitis; **TNF =** tumor necrosing factor.

Regarding to biological therapy, Paschou et al. (7), in 2009, assessed AS patients who were treated with infliximab and reviewed their medical records. They found that all of them had successfully fathered at least one children even one patient that also received small doses of methotrexate. Reinforcing this finding, our group evaluated 20 AS patients under TNF blockage and compared to 24 normal controls and showed no difference in sex hormone, inhibin B levels and seminal analysis (23).

### Behçet disease

There were six papers addressing BD and its relationship with male fertility. They are summarized in Table-5. An early report brought attention to possible side-effects of colchicine used in BD management with oligospermia in 11 of 136 patient’s (24). Later, Sarica et al. (25) evaluated 62 men with BD treated only with colchicine and evidenced oligonecrospermia in 37% and azoospermia in 3%. However, Fukutani et al. (26) evaluating 27 BD patients did not observe impact seminal parameters or FSH levels in patients treated with colchicine use alone.

**Table 5:** Behçet disease, gout and male fertility.

Author	Year	Study Population	Results	Conclusion
Behçet disease				

Mizushima et al. (24)	1977	157 patients with BD in colchicine use	11 patients had oligospermia	Low side-effects of colchicine use
Fukutani et al. (26)	1981	31 male patients with BD divided in four groups regarding medication use	Only the patients treated with CYC had seminal abnormalities and diminished FSH serum levels	BD did not impair testicular function / Testicular damage related to CYC
Tabbara (27)	1983	10 men with BD treated with chlorambucil	7 patients had oligospermia and the other 3 azoospermia	Chlorambucil should not be used as the first line of therapy in BD
Sarica et al. (25)	1995	62 male patients under colchicine therapy for BD	23 patients (37%) had oligonecrospermia and 2 patients (3%) azoospermia	Urological manifestation of BD and medication adverse reaction should be careful monitored
Shiraishi et al. (17)	2008	32 men with RA, 14 with BD, 8 with SLE and 80 healthy controls	None of BD patients had ASA	BD seems not to be related to ASA
Uzunalan et al. (28)	2013	162 men with BD, 48 with FMF, 79 with AS and 43 healthy controls	23 BD patients had fertility issues, most commonly varicocele / No difference in pregnancies or children conceived	BD does not significantly decrease patient’s fertility

Gout				

Yu (29)	1982	518 gout patients treated with colchicine	No fertility issues reported	Neither gout nor colchicine use impacts fertility

**Note: BD =** Behçet disease; **FSH =** follicle-stimulating hormone; **CYC =** cyclophosphamide; **AS =** ankylosing spondylitis; **ASA =** antisperm antibody; **FMF =** familial mediterranean fever

Alkylating agents may induce sperm abnormalities in BD patient’s, as reported in ten men using chlorambucil that had impairment of semen production: 7 with oligospermia and 3 with azoospermia (27).

A recent study evaluated fertility outcome of a larger retrospective cohort of BD patient’s compared to a control Group (28). They observed that 23 out of 130 subjects had infertility and the most common etiology was varicocele. In contrast, none of the 14 men with BD assessed for ASA had antisperm antibodies (17).

### Gout

There is only one report addressing gout and fertility in males (Table-5). In a large study with 540 young patients with gout, fertility status was preserved in patients treated with colchicine and none presented fertility issues during 20 years of follow-up (29).

## DISCUSSION

SLE is an uncommon disease in men. It affects males in a sex ratio of 1:9 (30), suggesting that sex hormones could modify susceptibility or reduce the expression of SLE (11). Infertility is an important issue for them nowadays due to better prognosis and quality of life. Six publications reported impaired testicular function and decreased semen production in SLE patient’s and their possible association with disease and treatment (11-14, 31, 32). The underlying mechanism for disease induced damage to the testis is not completely elucidated although some authors showed that there is immunopathologic lesion of the testis (and excurrent ducts) occurring through T cell-mediated mechanisms triggered by antigens or pathogens that disrupt testicular immune privilege (33, 34). Testicular aggression by alkylating agents has been described since 1972 showing that it impairs the sperm production presenting spermatogenesis abnormalities and Sertoli cell dysfunction (35-37).

Although semen analysis is considered the hallmark of male infertility evaluation, standard seminal parameters do not detect abnormalities in up to 20% of subfertile males (38). The routine measurements do not reveal seminal defects at molecular levels that might be induced by reactive oxygen species, which are associated with male infertility (39, 40). The 2010 World Health Organization guidelines have reduced the reference limits for seminal parameters and fail to satisfy clinical and statistical standards and pose the risk of misclassifying a subject’s true fertility status (41, 42). The introduction of new biomarkers of spermatic function in the clinical practice will allow a more precise evaluation of the real impact of rheumatic disease on male fertility potential. Unfortunately, to date, there is no available information in the literature and these aspects were not covered in the present review.

Autoimmunity also affects fertility by the presence of ASA. Immunologic infertility is characterized by the presence of antibodies against spermatozoa in the serum and/or in the seminal plasma or on the sperm surface (43). The presence of multiple ASA can lead to the immobilization and/or agglutination of spermatozoa, which blocks sperm-egg interaction. They can also prevent implantation or arrest embryo development (44, 45). In SLE patient’s ASA have been found in up to 42% of the patient’s. However, the real significance of ASA in infertile men is controversial and currently there is no standardized treatment regimens (46). Lastly, aneuploidies are frequent and may also contribute for fertility impairment in SLE patient’s; therefore kariotype should be evaluated to complete the fertility analysis of these patient’s, especially in those with severally compromised spermatogenesis (47).

The incidence of DM is 1.5 to 0.7 per 100.000 per person-years and there is a trend to affect more women than males in a 1.9 ratio (48, 49). The fertility evaluation of this particular Group has limited publications. The most important contributing factors of infertility in male DM are disease activity and CYC use (6), generally associated with high doses of this alkylating agent (5, 50).

Several large Scandinavian cohorts and a cohort study in the United States demonstrate that women with RA have smaller families and are slower to conceive compared with other women (51). Even though female infertility has been extensively explored in past years, the evaluation of male fertility was overlooked. The few publications available show gonadal impaired function with elevated LH/FSH, patients with difficult to conceive and also a higher incidence of ASA (17, 21). A more extensive evaluation of this set of patients is necessary to have a clear sense of RA real impact on male fertility.

Most physicians agree that diseases such as RA and AS can cause significant limitations in sexual activity due to diminished desire and impaired mechanical capacity (52, 53). In spite of that, evaluation of sex hormonal levels, seminal analysis and varicocele have shown that AS disease and treatment do not have a major impact in male fertility (7, 21-23).

BD is a multisystemic vasculitis with musculoskeletal, muco-cutaneous, ocular, gastrointestinal and neurological findings. The disease is frequently seen in the Mediterranean basin and the Far East. Young adults in their 20s and 30s are typically affected, during their reproductive years, with no gender predilection. However, males usually have a more severe course of the disease and are prone to present eye and other major organ involvement (28). The available literature about BD relationship with infertility is not robust. The disease itself seems to have no impact on fertility potential, but alkylating agents use is associated with its decrement (26, 27). The aggression to testicular tissue by colchicine reported by some authors in BD (25) was not confirmed in a large gout study (28).

Gout is induced by the deposition of monosodium urate crystals in synovial fluid and other tissues associated with hyperuricemia (54). Patient’s with gout are usually older and fertility often is not an issue, as most of them had already constituted their families. Although there is a concern about colchicine impairing reproductive function, decreased fertility has not been found in these patient’s (29).

And finally, the modern view of male fertility evaluation gives a new meaning to the term “male infertility management”, which goes beyond the simple identification and elimination of the cause. The andrologist’s therapeutic strategies have changed from a recent past of only attempting to achieve a simple increase in the sperm concentration. We are moving forward and now our main target is to improve the “quality” of spermatozoa and/or preserve fertility (55). This approach is thus especially recommended for patients with rheumatic diseases, often not considered potentially fertile.

## CONCLUSIONS

Rheumatic diseases comprise a heterogeneous Group of diseases with distinct aspects regarding male infertility. SLE clearly affects gonadal function impairing spermatogenesis due to ASA and CYC therapy. Fertility seems to be not affected in BD and AS patients, including patients under anti-TNF therapy. The fertility potential of DM patients may be affected by the disease activity and by alkylating agents. There are also few data regarding RA, however male gonad may be affected by the disease activity and ASA. Gout patients usually do not have any conception desire at the time of disease manifestation and there are no reports of fertility impairment.

A multidisciplinary Group is essential to assess male reproductive health in rheumatic disease patients with a special focus on improving the fertile potential and sexual dysfunction to minimize the disease and treatment damage.
